# Transmission pathways during high-contact care activities in nursing homes: a high-fidelity simulation study with surrogate markers

**DOI:** 10.1017/ice.2026.10431

**Published:** 2026-05

**Authors:** Paige R. Gannon, Victoria R. Dotto, Kylie B. Burke, Rachel Regina, Jesse T. Jacob, Joel M. Mumma

**Affiliations:** Division of Infectious Diseases, Emory University School of Medicine, USA

## Abstract

**Objective:**

To understand how the workflows and infection prevention and control (IPC) practices of certified nursing assistants (CNAs) during high-contact resident care activities contribute to multidrug-resistant organism (MDRO) transmission in nursing homes.

**Methods:**

We conducted 10 high-fidelity simulations of two high-contact resident care activities, bed bathing and incontinence care, with CNAs from long-term or mixed-care units. Four genetic variants of λ phage were applied to select surfaces prior to simulations and subsequently sampled from the environment, residents, supplies, and CNAs. Simulations were video recorded and analyzed for patterns of hand-to-surface contact and performance of IPC practices, including hand hygiene, personal protective equipment use, and environmental surface cleaning or disinfection.

**Results:**

A median of 11.5 transmission events occurred per simulation. Most events (60%) occurred within residents’ immediate environments, reflecting how CNAs frequently transitioned between a resident, their surroundings, and care supplies, combined with infrequent hand hygiene and surface disinfection. Contamination of CNA scrubs and hands accounted for 24% of events, primarily from bed bathing, which involved frequent contact without a gown. Transmission to shared objects (e.g., linen bin, trash can, wheelchair) accounted for 16% of events and created additional opportunities for transmission between residents. Transmission between residents or their immediate environments was rare but typically associated with workflow disruptions from limited-supply availability.

**Conclusions:**

In high-fidelity simulations of high-contact resident care activities, transmission of surrogate markers for MDROs closely followed the workflows of CNAs. This method identifies potential transmission pathways and interventions for mitigating MDRO spread in nursing homes.

## Introduction

In nursing homes, infections are a major cause of resident morbidity and mortality.^1^ Over half (57%) of nursing home residents are colonized with a multidrug-resistant organism (MDRO), including methicillin-resistant *Staphylococcus aureus* (MRSA), vancomycin-resistant enterococci (VRE), and extended-spectrum β-lactamase (ESBL)-producing organisms.^2,3^ MDROs can spread when healthcare workers (HCWs) acquire contamination on their hands or clothing through physical contact with a resident who is colonized or infected and subsequently transmit it to other residents.^4,5^ Many activities of daily living (ADLs) involve close, prolonged contact between HCWs, residents, and their immediate surroundings. Consequently, these particular activities pose a high risk of HCWs’ hands or clothing becoming contaminated with or transmitting MDROs, such as MRSA or ESBL-producing organisms.^6–9^

Prior research has identified common challenges that contribute to the risk of transmission during high-contact activities, such as bathing or changing resident briefs.^5,10^ These challenges arise from multilevel factors, including resident-related factors (e.g., immobility, incontinence, aggression) and organizational constraints (e.g., inadequate staffing, limited or poor-quality supplies). Such challenges can disrupt HCWs’ workflow or undermine their adherence to infection prevention and control (IPC) practices such as hand hygiene, use of personal protective equipment (PPE), and environmental surface cleaning or disinfection. However, understanding how these challenging workflows influence transmission during resident care remains limited, yet it is important for identifying barriers to existing IPC practices or where new interventions may be needed.

This study aimed to characterize certified nursing assistant (CNA) workflows and IPC practices during two high-contact resident care activities and examine how these behaviors may contribute to MDRO spread within nursing homes. CNAs were the focus of the present study because they provide the majority of direct resident care, including high-contact activities such as bathing and brief changes.^11^ To this end, we conducted high-fidelity simulations of resident care with CNAs using a bacteriophage as a surrogate marker for MDRO transmission, combined with behavioral analyses of simulation recordings.

## Methods

### Participants

Between August and November 2023, we conducted 10 standardized resident care simulations with CNAs who worked in long-term care or mixed-care units (e.g., residents receiving long-term or skilled nursing) in a nursing home. Participants had a median of 21 years (IQR = 17.2–28.3) of experience as a CNA and worked on either a long-term (50%) or mixed-care (50%) unit. Participants were compensated with a $25 gift card and a parking voucher for their time. All research activities were approved by Emory University’s Institutional Review Board (STUDY00004038).

### High-fidelity simulation scenario

**Figure 1.**
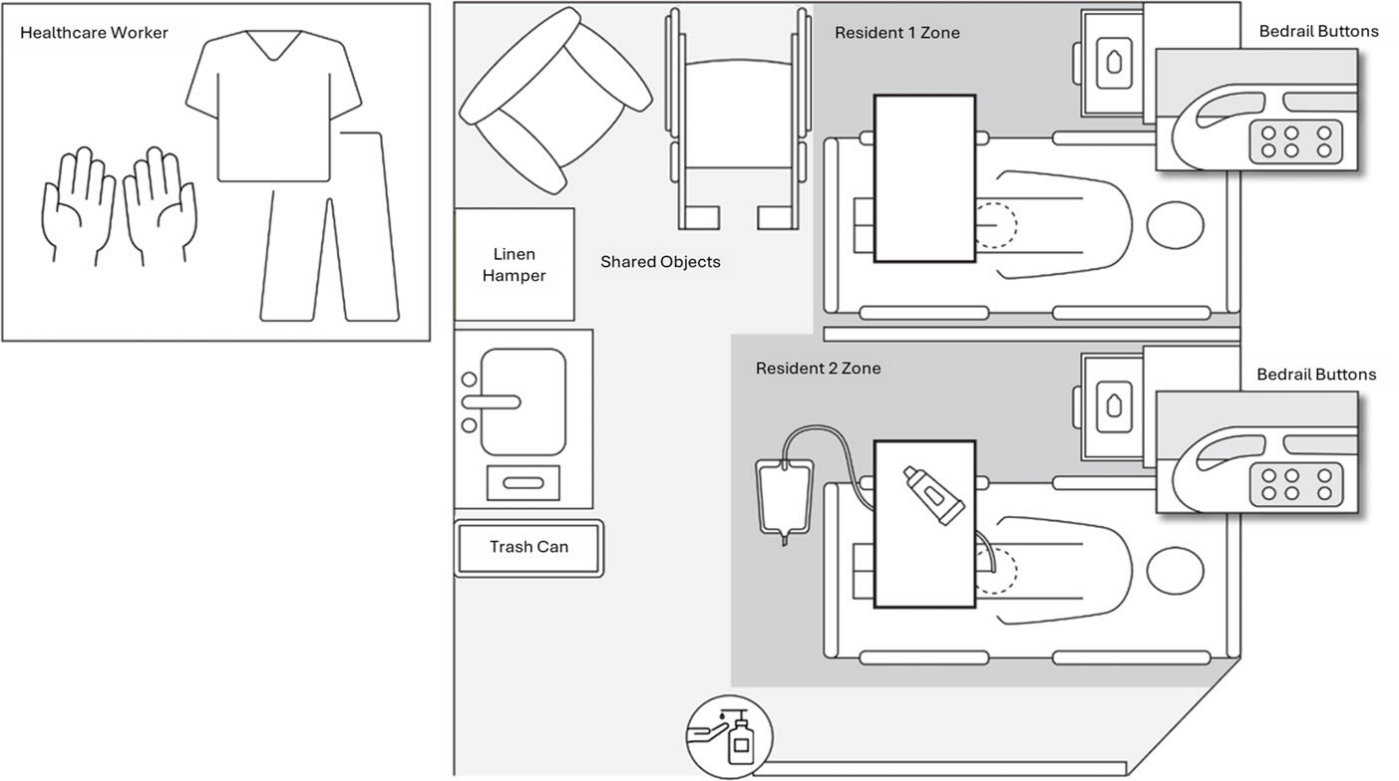
Layout of a simulated double occupancy room in a nursing home.

We developed a high-fidelity simulation scenario of high-contact resident care activities involving two adult male nursing home residents (Figure 1). The residents, represented by mannequins, shared a single room with a curtain dividing their immediate surroundings (“zones”), a common configuration in nursing homes. Resident 1 was incontinent of urine, had simulated skin breakdown in the groin area, and needed his brief changed and skin barrier cream applied to the skin breakdown. Resident 2 was incontinent of stool, had an indwelling urinary catheter, and needed a complete bed bath and linen change. Options for hand hygiene included alcohol-based hand rub dispensers located at the entryway of the resident’s room or a sink with hand soap in the middle of the shared room. Gloves were available on the sink in the resident’s room, while isolation gowns and containers of disinfecting wipes, which are typically stored outside of resident rooms,^12^ were available in a separate supply room. A trash can and a linen hamper were located next to the sink.

Based on prior qualitative research with CNAs, the simulation scenario also included common challenges to performing care activities that may contribute to transmission by promoting lapses in IPC practices or increasing the amount of physical contact between the CNA, residents, and their environment.^10^ These challenges in the simulation included limited availability of resident care supplies (e.g., skin barrier cream), environmental clutter (e.g., furniture, a wheelchair), having residents with fecal or urinary incontinence, and residents who require total care (i.e., are completely dependent on staff for ADLs).

### Procedure

Before each simulation, four genetic variants of *λ* phage were applied to predetermined sites on residents’ bodies and environmental surfaces in the simulation space. Resident 1 had *λ* ^Temp^ applied to simulated skin breakdown in the groin area and the incontinence brief and *λ* ^Kan^ on the front bedrails and bedside table. Resident 2 had *λ* ^Vir^ in simulated stool that was in their brief, on their indwelling urinary catheter, on their right back bedrail, and on one side of the bedside table. *λ* ^Chl^ was applied to Resident 2’s front bedrails and the side of the bedside table that was not contaminated by *λ* ^Vir^. Each λ variant was applied with a spray bottle containing 10 mL of a 10^8^ plaque-forming units (PFU)/mL solution.^13^

Upon arrival, participants were consented and completed a demographic questionnaire. Research staff then oriented the participant to the simulation space, supplies, and resident care activities. Participants then donned a pair of disposable scrubs and a head-mounted camera (GoPro®). The simulation began after the research staff gave a scripted handover for each resident to the participant, who could choose which task and resident to begin with. Participants were told that contamination was present in the simulation but were not aware of its locations.

After each simulation, 24 predetermined sites were sampled for the presence of each *λ* variant (Table 1). These surfaces comprised environmental surfaces (n = 10 sampling sites), resident sites (n = 5), task supplies (n = 3), shared objects (n = 3), and surfaces on participants (n = 3). After sampling, surfaces were disinfected using 70% ethanol, DNA Away™, RNase Away™, and Lysol® wipes. The identity of λ variant(s) from positive surface samples was determined through direct polymerase chain reaction testing. A “transmission event” occurred when a *λ* variant was detected on a sampling surface on which that variant was not applied prior to the simulation (Table 1). Detailed methods for contamination, surface sampling, and decontamination procedures are described and validated elsewhere.^13^

**Table 1.** Sampling surfaces and sources of contamination in each simulation

Surface type	Resident	Sampling location(s)
Environmental surface	1	Bedside table,^b^ back bedrails, front bedrails,^b^ bedrail buttons, nightstand handles
	2	Bedside table,^c,d^ back bedrails,^c^ front bedrails,^d^ bedrail buttons, nightstand handles
Resident site	1	Skin breakdown/groin,^a^ body
	2	Indwelling urinary catheter,^c^ body, groin
Task supply	1	Package of resident cleansing wipes
	2	Tube of skin barrier cream, package of resident cleansing wipes
Shared object	N/A	Linen hamper, trash can, wheelchair
Healthcare worker	N/A	Scrub top, scrub bottom, bare hands

N/A, not applicable.

^a-d^Sampling locations inoculated with λ^Temp^ (a), λ^Kan^ (b), λVir (c), or λChl (d) prior to the start of each simulation.

### Behavioral coding

We developed a behavioral coding scheme to capture sequences of hand-to-surface contacts and performance of IPC practices in video recordings of simulations (see Supplemental Material). Contact with a surface was coded whenever a participant grabbed or held a new surface with either hand but was not coded when repeatedly touching the same surface or when transferring the same object between hands. If a participant grabbed or held multiple new surfaces simultaneously, contact with all surfaces was coded. For hand hygiene, we recorded whether participants used alcohol-based hand rub or soap and water. For glove use, we coded when both gloves were fully donned or doffed. For surface cleaning and disinfection, we coded each time an object or surface was wiped and whether participants used a resident cleansing wipe, a washcloth or towel, or a disinfecting wipe.

After developing the initial behavioral coding scheme, three researchers independently coded one pilot simulation video. The researchers then discussed disagreements, revised the coding scheme, and independently coded a video of a different pilot simulation. Interrater reliability was acceptable (Cohen’s *κ* = 0.68; 95% CI: 0.64–0.71), and each researcher subsequently coded 3–4 simulation videos. Behavioral coding was performed using The Observer XT software.^14^

### Workflow analysis

To characterize CNA workflows in the simulations, we conducted a two-phase analysis of the behavioral coding data. In the first phase, we performed a lag sequential analysis (LSA) using The Observer XT software.^14^ LSA calculated the total frequency at which contact with each object (or performance of an IPC practice) immediately followed contact with every other object or IPC practice in each simulation. Before performing LSA, data were preprocessed by consolidating interactions with objects that had similar potential for hand-to-surface transmission. For example, both grabbing a bedside table and cleaning it with a resident cleansing wipe were counted as contact with the bedside table. In contrast, the use of disinfecting wipes on a surface was not counted as contact, given their ability to eliminate pathogens, including bacteriophage.^13^

In the second phase of analysis, we examined the structure of CNA workflows using the Walktrap community detection algorithm^15^ in the *igraph* package in *R*.^16^ The Walktrap algorithm identifies densely connected communities of nodes (i.e., objects or IPC practices) by simulating short, random walks on a network where the probability of transitioning between two nodes is proportional to the weight of the edge connecting them.^15^ In terms of workflows, communities are groups of contiguous behaviors, which may correspond to distinct tasks or subtasks. To apply the Walktrap algorithm, we first converted the output of the LSA into an undirected, weighted graph by retaining edges between objects and IPC practices only if transitions occurred in both directions at least once. The weight of each edge was then calculated as the sum of transitions in both directions.

## Results

### Workflow analysis

Simulations lasted a median of 38.5 minutes (IQR: 32.5–46). Across the 10 simulations, there were 3,083 hand-to-surface contacts (median = 282.5 contacts per simulation, IQR: 228.75–378). Most contacts (78%, 2,398/3,083) occurred within resident zones (i.e., a resident and their immediate surroundings; Figure 1), with contact within Resident 2’s zone alone accounting for 58% (1,783/3,083) of all contacts. Resident 2, who required a complete bed bath and linen change, was also the single most frequently contacted object (Median = 33.5 contacts per simulation, IQR: 24.25–47.5).

**Figure 2.**
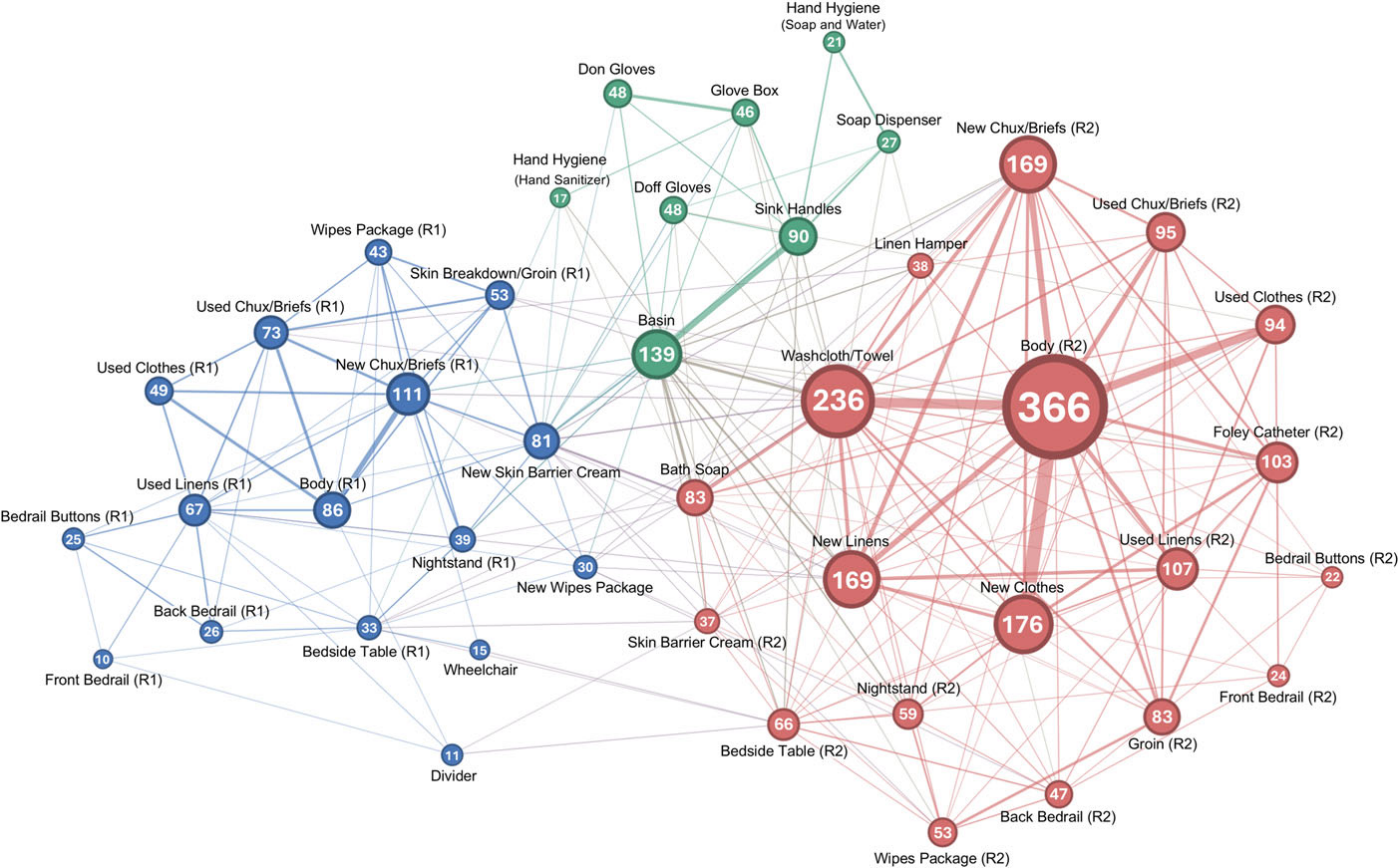
Weighted, undirected graph of transition frequencies between objects or IPC practices (nodes). Node values and edge thickness represent the total frequency of transitions to and bidirectional transitions between nodes, respectively. The trashcan was excluded due to no bidirectional transitions. Abbreviations: R1, resident 1; R2, resident 2.

Most CNAs (80%, 8/10) began the simulation with Resident 1, who required a brief change and application of skin barrier cream to skin breakdown on his groin. CNAs’ workflows were otherwise highly modular, forming three distinct communities: one community encompassed all objects in Resident 1’s zone (blue; Figure 2), another all objects in Resident 2’s zone (red), and a third (green) comprising IPC practices (e.g., hand hygiene, glove use) and objects needed to perform them (e.g., sink, glove box). Communities also included new supplies from the supply room or shared objects that were closely associated with a resident and their activities, such as the linen hamperin Resident 2’s community. The separation of IPC practices indicates that these activities were performed outside of, rather than during episodes of resident care, while the separation between resident communities reflects how CNAs tended to complete all care for one resident before beginning care for another. Within resident communities, CNAs frequently transitioned between touching the resident, their immediate surroundings, and task supplies.

### Transmission events

**Figure 3.**
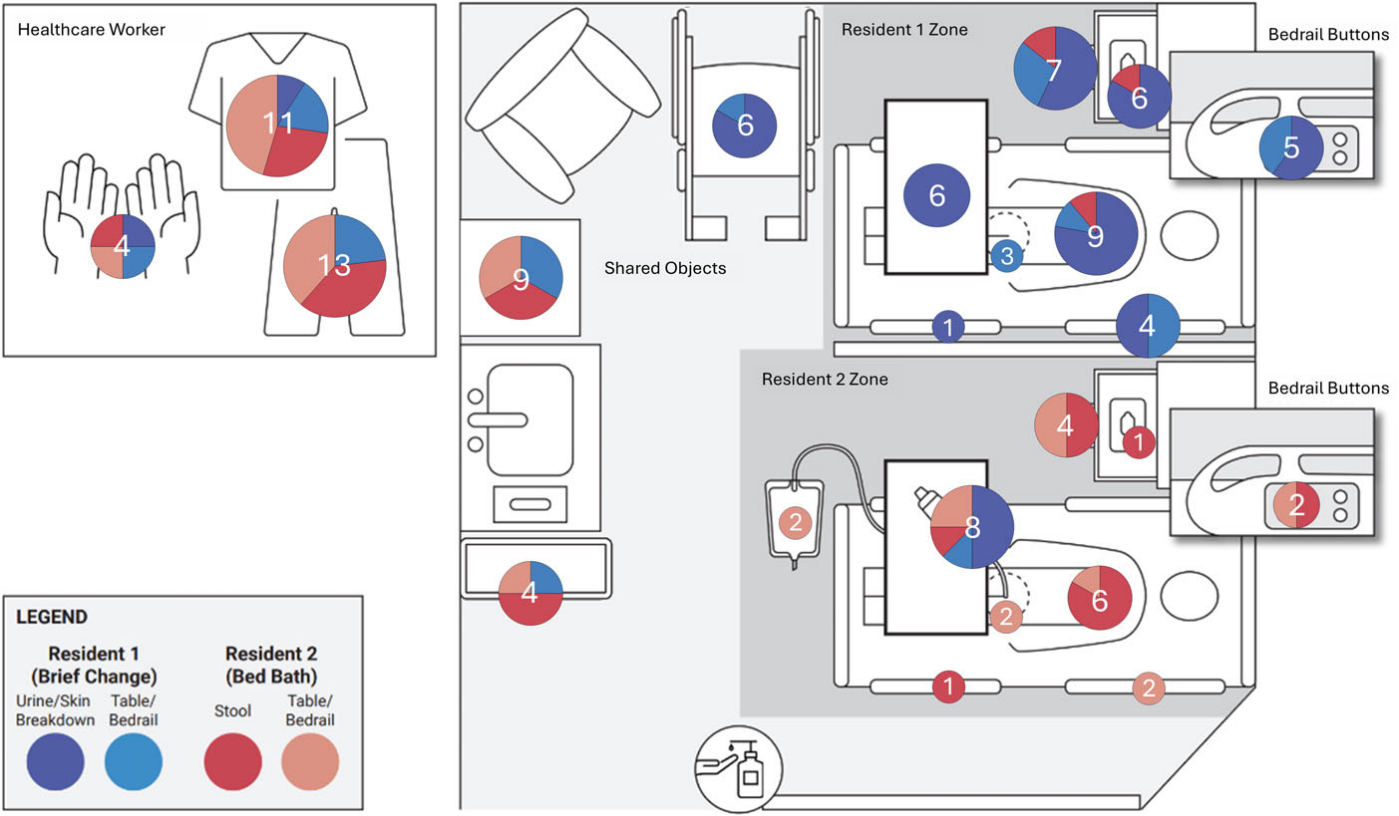
Pie charts show the total number of transmission events on a sampling surface (in white) and the proportion of transmission events on sampling surfaces from each λ variant.

Across the 10 simulations, there were 116 transmission events (median = 11.5 events per simulation, IQR: 7.25–15.75). Most events (60%, 69/116) occurred within resident zones (Figure 3) and overwhelmingly involved transmission of a λ variant originating from that zone (88%, 61/69). Transmission between resident zones was less common (12%, 8/69), occurring primarily in Resident 2’s zone and involving transmission from Resident 1’s skin breakdown to Resident 2’s skin barrier cream, which was a limited supply in the simulation.^10^

Surfaces on CNAs, such as their scrubs and bare hands, accounted for 24% (28/116) of transmission events and had the highest contamination rate of all types of sampling surfaces (median = 2 events per simulation, IQR: 2–3.75). Most transmission to CNAs was to their scrubs (86%, 24/28), which originated mostly from Resident 2 (71%, 20/28).

Lastly, transmission to shared objects, such as the linen hamper and trash can, accounted for 16% (19/116) of events. Transmission to these objects originated from Residents 1 and 2 to similar degrees (53% [10/19] versus 47% [9/19], respectively) except for the wheelchair, which only had contamination from Resident 1.

### Infection prevention and control practices

CNAs performed hand hygiene a median of 3.5 times per simulation (IQR: 2–5.75), using soap and water and alcohol-based hand rub in 55% (21/38) and 45% (17/38) of instances, respectively. Hand hygiene was most often performed at the start of the simulation (29%, 11/38), after completing care for one resident but before beginning care for the next (29%, 11/38), after completing care for both residents (26%, 10/38), and least often, during resident care itself (16%, 6/38).

Regarding PPE use, CNAs donned and doffed gloves a median of 5 times per simulation (IQR: 3–6), performing hand hygiene either before donning or after doffing gloves in 42% (20/48) of instances, both before donning and after doffing (8%, 4/48), and never (50%, 24/48). No participant wore an isolation gown despite them being available in the supply room.

Lastly, CNAs attempted to clean or disinfect environmental surfaces a median of 4 times per simulation (IQR: 2.25–5), using non-disinfecting wipes in 82% (39/48) of instances, which included resident cleansing wipes or “improvised” wipes made from a paper towel and hand sanitizer, washcloths in 10% (5/48) of instances, and disinfecting wipes in 8% (4/48) of instances.

## Discussion

This study used high-fidelity simulations and surrogate markers to examine how contamination spreads during high-contact resident care activities in nursing homes. Contamination of HCWs, residents, their immediate environments, and shared objects was common. Behavioral analyses indicated that transmission is shaped not only by adherence to IPC practices but also by the organization and flow of resident care activities.

Most transmission events involved the movement of phage within a resident’s zone, which reflected how CNAs frequently transitioned between touching a resident, their immediate surroundings, and care supplies during care activities. IPC practices, such as changing gloves, hand hygiene, and surface cleaning or disinfection occurred infrequently, leaving few barriers to interrupt transmission within resident zones. When surface cleaning or disinfection did occur, it was rarely performed with a disinfecting product; in nursing homes, cleaning products are stored outside resident rooms to prevent misuse by cognitively impaired residents, a practice that also limits staff access and use during care.^12^ By increasing environmental bioburden, transmission within resident zones creates opportunities for re-contaminating a resident (e.g., colonizing a new body site) or transmission outside a resident’s zone.^17–19^

Transmission outside of resident zones primarily involved surfaces that could facilitate spread between residents. Shared objects, such as trashcans, linen hampers, and wheelchairs, can indirectly transmit contamination through repeated handling by HCWs and use across multiple residents. Wheelchairs, in particular, can be an overlooked vector for transmission due to their frequent use and inconsistent disinfection between residents.^20,21^ In addition to shared objects, CNAs’ scrubs were frequently contaminated, especially during bed bathing of Resident 2. This finding aligns with prior evidence identifying HCW clothing as a vector for MDRO transmission.^22–24^ Behavioral analyses suggested two contributing factors: a high amount of physical contact during bed bathing and the absence of gown use, despite their availability. These findings reinforce the importance of targeted gown and glove use during high-contact care activities, as recommended in the Centers for Disease Control and Prevention’s concept of Enhanced Barrier Precautions (EBP).^5,25^ Additionally, other strategies to reduce contact during bed bathing should be considered. For example, alternatives to bathing, such as using no-rinse bath wipes, may decrease the intensity and duration of physical contact during bed bathing.^26^

Transmission between resident zones was rare and appeared to be related to the larger structure of workflows. CNAs typically completed all care activities for one resident before moving to the next, which limited opportunities for direct transmission between residents. When transmission did occur, it was mainly from Resident 1 to Resident 2 and was associated with having limited resident care supplies.^10^ Most CNAs began the simulation with Resident 1, who needed a brief change and skin barrier cream applied to the skin breakdown on his groin. Although cream was stocked in the supply room, it was only immediately available on resident 2’s bedside table. Retrieving the supply during Resident 1’s care may have disrupted the CNAs’ workflow and contributed to lapses in IPC practices at this critical moment.^10^

This study has several limitations. First, the small number of participants could not capture the full variability of workflows and adherence to IPC practices in nursing homes. Second, we assessed positivity rather than density of recovered phage from surfaces within a resident’s room as our focus was on identifying potential transmission pathways rather than quantifying the risk associated with these pathways. Third, participants were aware that they were being recorded during their simulations, which may have influenced their behavior. Lastly, we conducted the study before the 2024 Center for Medicare and Medicaid Services mandate for EBP in nursing homes, and outcomes, such as frequent scrub contamination, may have differed had EBP been followed in the simulations.

Overall, our findings suggest a close relationship between HCW workflows and transmission during high-contact resident care activities. Targeted interventions include providing adequate and accessible supplies,^10^ altering tasks to reduce HCW contamination (e.g., alternative approaches to bed bathing), improving hand hygiene and PPE use at the bedside (e.g., EBP), and environmental surface disinfection of shared objects and high-touch surfaces. High-fidelity simulation with surrogate markers, combined with behavioral analysis, is useful for identifying transmission pathways during resident care activities, and future research should use this method to evaluate potential interventions.

## Supporting information

Gannon et al. supplementary material Gannon et al. supplementary material

## Supplementary material

The supplementary material for this article can be found at https://doi.org/10.1017/ice.2026.10431.
