# Supplementary material for: Transmission pathways during high-contact care activities in nursing homes: a high-fidelity simulation study with surrogate markers
Source: Infect Control Hosp Epidemiol. 2026 Mar 26;47(5):470–5. doi: 10.1017/ice.2026.10431 (PMC13038848; doi:10.1017/ice.2026.10431)
Supplement: Gannon et al. supplementary material [file S0899823X26104310sup001.docx]

**Behavioral Coding Scheme Rules:**

1. Starting Codes:
   1. Simulation coding starts at the beginning of handoff.
   2. Whenever either hand interacts with a new object, it is a new code (A; **Supplementary Figure 1**). If the other hand then interacts with the same object, it is not a new code (B). If the original object then transfers hands after touching an intervening object, it is a new code (C).


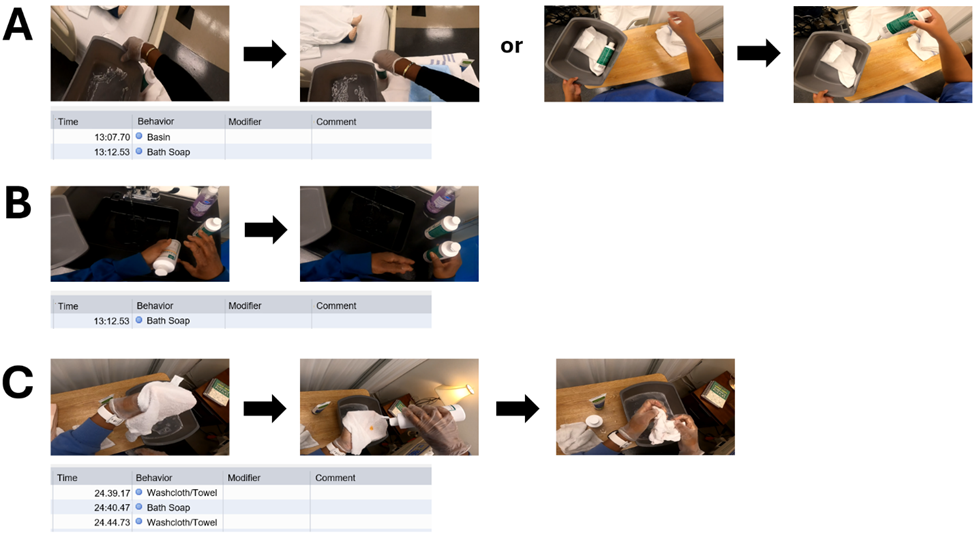


**Figure 1.** Illustration of when to code a new hand-to-surface contact.

- 1. Grazing an object without grabbing or holding the object is not a new code.
  2. If touching or grabbing multiple objects or surfaces at the same time, code all objects and surfaces.
  3. Codes for objects and their associated modifiers are provided in **Supplementary Table 1** and **Supplementary Table 2**.

1. Infection Prevention & Control Practices:
   1. **Hand Hygiene (HH):** Starts the first time the hands come together and ends the last time the hands come apart before touching an object or surface. In the comments, mark the duration that hands are actively rubbed together.
      1. HH not coded when:
         1. HH on only one hand.
         2. HH with only use of water or only use of soap.
         3. HH on gloves.
         4. HH continues after interacting with an object.
      2. *Modifier (Hand Hygiene)*: Designate what method HH was performed with (soap/water or hand sanitizer)
   2. **Gloves:** Only code "Don" when both gloves are on and "Doff" when both gloves are off.
      1. Glove use not coded when:
         1. Only one hand is donned.
         2. Double gloving.
      2. *Modifier (Gloves)*: Designate donning and doffing.
   3. **Cleaning/Disinfecting Surfaces:** Used as an optional modifier when cleaning a surface. For each surface that is cleaned, even with the same wipe, it is coded as a new behavior.
      1. *Modifier (Type of Wipe)*: Designate what type of wipe was used.
2. Object Specific Codes:
   1. **Washcloths/towels (terrycloth):** A washcloth or towel is coded when either of these objects are interacted with outside of cleaning (e.g., gathering wash cloth or towel from supply room).
   2. **Linens:** Each new linen touched is coded as a separate behavior.
      1. *Modifier (Sate)*: Designate if linen is New or Used based on starting location.
   3. **Clothes:** Used when coding resident gowns or their clothing.
      1. *Modifier (State)*: Designate if clothes is New or Used based on starting location.
   4. **Chux/briefs:** Each new chux or brief they touch is coded as a separate behavior.
      1. *Modifier (State; Resident)*: Designate if chux or brief is New or Used and associated Resident based on starting location.
   5. **Resident Cleansing Wipes Package:**
      1. *Modifier (Wipes)*: Designate New or associated Resident based on starting location.
   6. **Skin Barrier Cream:**
      1. *Modifier (Cream)*: Designate New or Resident 2 skin barrier cream.
   7. **"Other":** If an object is touched outside of the set list, describe what that object is in the comment.
3. Other Coding Behaviors:
   1. If a surface is fully covered with an object, and the surface is then moved, only code the object covering the surface.
      1. Ex. A bedside table is covered with a towel. If the bedside table is moved, but only the towel covering the table is touched, code as “Washcloth/Towel”.
   2. If the groin is wiped with a used brief, code as “Used Chux/Brief” with the appropriate patient modifier.
   3. If one glove is removed and passed between hands (one gloved and one not gloved), continue to follow coding scheme as normal, do not code gloves as an object.

**Supplementary Table 1.** Codes and their associated modifiers.

| Code | Modifier |
| --- | --- |
| *General Objects* |  |
| Wheelchair | N/A |
| Trash Can |  |
| Linen Hamper |  |
| Glove Box |  |
| Divider |  |
| Soap Dispenser |  |
| Sink Handles |  |
| Basin |  |
| Bath Soap |  |
| Washcloth/Towel |  |
| Other (Describe) |  |
| Linens | State |
| Clothes |  |
| Chux/Briefs | Resident, State |
| Skin Barrier Cream | Cream |
| Wipes Package | Wipes |
| *Resident Objects* |  |
| Bedrail Buttons | Resident, Type of Wipe |
| Bedrail Back |  |
| Bedrail Front |  |
| Bedside Table |  |
| Nightstand |  |
| Body |  |
| Groin | Type of Wipe |
| Skin Breakdown |  |
| Foley |  |
| *Infection Prevention & Control Practices* | |
| Glove | Gloves |
| Hand Hygiene | Hand Hygiene |

See Supplementary Material Table 2 for modifier options and definitions.

Abbreviations: N/A, not applicable

**Supplementary Table 2.** Coding modifiers, modifier options, and their definitions.

| Modifier | Modifier Options | Modifier Definition |
| --- | --- | --- |
| Resident | Resident 1 | Used when describing an object belonging to Resident 1 or their body. |
|  | Resident 2 | Used when describing an object belonging to Resident 2 or their body. |
| State | New | Object began in the supply closet and was not used as part of resident care until it was introduced by the CNA. |
|  | Resident 1 Used | Object was located among Resident 1’s possessions (i.e. in nightstand) or was in use by Resident 1 at the start of the simulation (i.e., bed sheets). |
|  | Resident 2 Used | Object was located among Resident 2’s possessions (i.e. in nightstand) or was in use by Resident 2 at the start of the simulation (i.e., bed sheets). |
| Type of Wipe | Washcloth/Towel | A washcloth or towel was used to wipe the body or surface. |
|  | Improvised Wipe | An improvised wipe (i.e., paper towel and hand sanitizer) was used to wipe the body or surface. |
|  | Resident Cleansing Wipe | A resident wipe (i.e., bed bathing wipe) was used to wipe the body or surface. |
|  | Disinfecting Wipes | A disinfecting wipe was used to wipe the body or surface. |
| Wipes | New | Wipes package originated from the supply closet and was not used as part of resident care until introduced by the CNA. |
|  | Resident 1 | Wipes package started in Resident 1’s nightstand drawer. |
|  | Resident 2 | Wipes package started in Resident 2’s nightstand drawer. |
| Cream | New Cream | Skin barrier cream originated from the supply closet and was not used as part of resident care until introduced by the CNA. |
|  | Resident 2 Cream | Skin barrier cream started on the bedside table of Resident 2. |
| Gloves | Don | Both gloves were put on the CNAs hands. |
|  | Doff | Both gloves were removed from the CNAs hands. |
| Hand Hygiene | Hand Sanitizer | Use of hand sanitizer to perform hand hygiene. |
|  | Soap and Water | Use of soap and water to perform hand hygiene. |

Abbreviations: CNA, Certified Nursing Assistant
